# Metastatic Pheochromocytoma/Paraganglioma Overproducing Multiple Catecholamines

**DOI:** 10.1210/jcemcr/luae241

**Published:** 2024-12-26

**Authors:** Keiko Yoshioka, Yujiro Nakano, Moeka Horichi, Daisuke Aono, Yumie Takeshita, Toshinari Takamura

**Affiliations:** Department of Endocrinology and Metabolism, Kanazawa University Graduate School of Medical Sciences, Kanazawa University, Kanazawa, 920-8641, Ishikawa, Japan; Department of Endocrinology and Metabolism, Kanazawa University Graduate School of Medical Sciences, Kanazawa University, Kanazawa, 920-8641, Ishikawa, Japan; Department of Endocrinology and Metabolism, Kanazawa University Graduate School of Medical Sciences, Kanazawa University, Kanazawa, 920-8641, Ishikawa, Japan; Department of Endocrinology and Metabolism, Kanazawa University Graduate School of Medical Sciences, Kanazawa University, Kanazawa, 920-8641, Ishikawa, Japan; Department of Endocrinology and Metabolism, Kanazawa University Graduate School of Medical Sciences, Kanazawa University, Kanazawa, 920-8641, Ishikawa, Japan; Department of Endocrinology and Metabolism, Kanazawa University Graduate School of Medical Sciences, Kanazawa University, Kanazawa, 920-8641, Ishikawa, Japan

**Keywords:** dopamine, metastatic PPGL, DBH, PNMT

## Abstract

Pheochromocytoma and paraganglioma (PPGL) are rare chromaffin-cell tumors producing adrenaline and/or noradrenaline, or solely dopamine. A 52-year-old man presenting with hypertension (141/79 mm Hg) and weight loss (10 kg in 6 months) was admitted to our hospital. Computed tomography revealed a massive right adrenal mass (150 mm) with partial necrosis, accompanied by multiple liver nodules. These nodules showed a high signal intensity on T2-weighted magnetic resonance imaging. Subsequently, a diagnosis of PPGL was made based on elevated urinary excretion of adrenaline (355 µg/day [1937 nmol/day]; normal range: 3.4-26.9 µg/day; 18-146 nmol/day), noradrenaline (1690 µg/day [9989 nmol/day]; normal range: 48.6-168.4 µg/day; 287-995 nmol/day), and dopamine (53 000 µg/day [258 322 nmol/day]; normal range: 365-961.5 µg/day; 1779-4686 nmol/day). The ^123^I-metaiodobenzylguanidine scintigraphy and fluorodeoxyglucose positron emission tomography scan showed heterogenous uptake among the adrenal and the liver foci, respectively. Clustering analysis of previous PPGL cases highlighted the unique catecholamine profile of this case. These findings suggest a possibility that internodular heterogeneity between primary and metastatic foci on nuclear imaging may indicate varying differentiation grades and resultant catecholamine secretion. Further studies will be needed to verify these results and confirm this hypothesis.

## Introduction

Pheochromocytoma and paraganglioma (PPGL) are rare chromaffin-cell tumors arising from the adrenal medulla and sympathetic or parasympathetic paraganglia, respectively. Most PPGL arising from the adrenal medulla and sympathetic paraganglia are associated with catecholamine excess, predominantly adrenaline or noradrenaline, that activate the respective adrenergic receptors, resulting in multitude of symptoms (headaches, hyperhidrosis, hypermetabolism, hyperglycemia, and malignant hypertension) [1]. In 10% of cases, PPGL cases metastasize to other organs, including the liver, resulting in a poor prognosis. The major cause of metastatic PPGL is a somatic or germline pathogenic variant in succinate dehydrogenase that mimics a hypoxia reaction and induces autonomous cell growth. However, genetic alterations vary among patients with PPGL [2].

**Figure 1.**
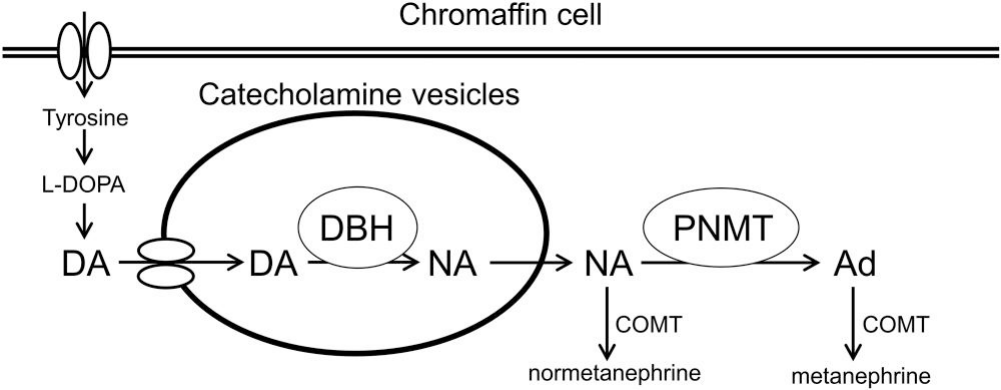
Schematic flowchart of major catecholamine synthesis and metabolism. Abbreviations: Ad, adrenaline; COMT, catechol-*O*-methyltransferase; DA, dopamine; DBH, dopamine β hydroxylase; NA, noradrenaline; PNMT, phenyl-ethanolamine N-methyltransferase.

Catecholamine synthesis depends on dopamine β hydroxylase (DBH) and phenyl-ethanolamine N-methyltransferase (PNMT). DBH and PNMT convert dopamine (DA) to noradrenaline (NA) and NA to adrenaline (Ad), respectively (Fig. 1). However, Ad is not produced in extra-adrenal PPGL due to absent PNMT, which is expressed in the adrenal medulla. In contrast, DBH is ubiquitously expressed in the adrenal and extra-adrenal chromaffin cells and contributes to NA production. DA-producing PPGL may be caused by deficiency or dysfunction of DBH and/or immature catecholamine vesicles, resulting in a marked elevation of DA compared to other catecholamines [3, 4]. There were few published cases reporting on elevation of DA and NA [4]. However, to date, none has described a concomitant elevation of DA, NA, and Ad levels in PPGL.

Here, we report a rare case of metastatic pheochromocytoma, with a right adrenal mass and multiple liver nodules, exhibiting overproduction of multiple catecholamines (DA, NA, and Ad). Previously published cases of DA-producing PPGL and patients with PPGL in our hospital were also reviewed in an attempt to further understand the characteristics of the present case. The findings presented for this case suggest the possibility that detailed examination is important in diagnosing and elucidating the mechanisms underlying metastatic PPGL.

## Case Presentation

A 52-year-old male was admitted to our hospital with the chief complaint of lumbar pain. Upon review of the patient, he endorsed hyperhidrosis and constipation, progressively worsening blood pressure over the past 3 years, and an unintentional weight loss of 10 kg in the preceding 6 months. Relevant family history was negative for PPGL. There was no history of medications such as carbidopa or levodopa that could cause elevation of dopamine levels.

## Diagnostic Assessment

**Figure 2.**
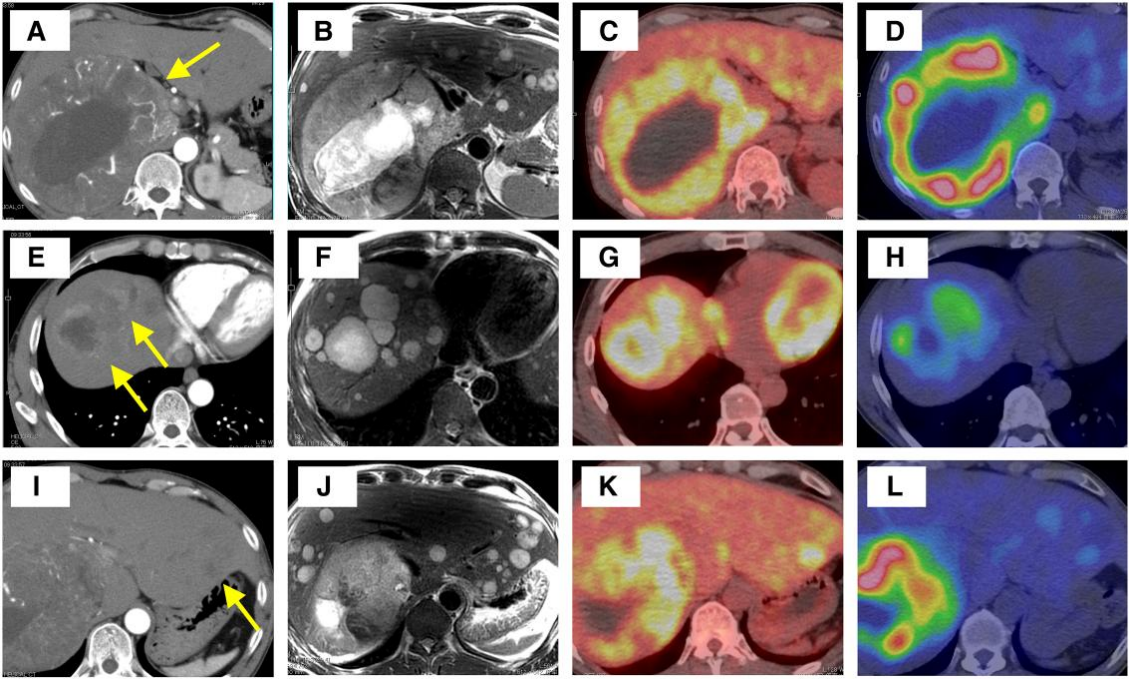
Imaging of adrenal and liver nodules. (A-D) Adrenal mass. (E-H) Liver nodule in the right lobe (S7, S8). (I-L) Liver nodules in the left segment (S2, S3). (A, E, I) Contrast CT. (B, F, J) MRI. (C, G, K) ^123^I-MIBG scintigraphy. (D, H, L) FDG-PET. Abbreviations: CT, computed tomography; FDG-PET, fluorodeoxyglucose positron emission tomography scan; MRI, magnetic resonance imaging; MIBG, metaiodobenzylguanidine.

The patient was 171.8 cm tall with a body weight of 68.7 kg, a blood pressure of 141/79 mm Hg, and a heart rate of 67 beats/min. The laboratory findings revealed mild liver dysfunction and elevated lactate dehydrogenase levels (Table 1). Computed tomography revealed a massive right adrenal mass (150 mm) with partial necrosis, accompanied by multiple liver nodules (Fig. 2). These nodules showed a high signal intensity on T2-weighted magnetic resonance imaging (Fig. 2). The urinary excretion of Ad, NA, DA, metanephrine, and normetanephrine was 355 µg/day (1937 nmol/day) (normal range: 3.4-26.9 µg/day; 18-146 nmol/day), 1690 µg/day (9989 nmol/day) (normal range: 48.6-168.4 µg/day; 287-995 nmol/day), 53 000 µg/day (258 322 nmol/day) (normal range: 365-961.5 µg/day; 1779-4686 nmol/day), 2.9 mg/day (14 703 nmol/day) (normal range: 0.0-0.19 mg/day; 0-963 nmol/day), and 8.6 mg/day (46 942 nmol/day) (normal range: 0.09-0.33 mg/day; 491-1801nmol/day), respectively (Table 2). ^123^I-metaiodobenzylguanidine (MIBG) and fluorodeoxyglucose (FDG) were strongly absorbed by the adrenal mass and slightly absorbed by the small liver nodules in S2 and S3 (Fig. 2). The liver nodules in S7 and S8 absorbed ^123^I-MIBG strongly and FDG partially (Fig. 2). The adrenocortical hormone levels were within normal ranges. The patient was subsequently diagnosed with metastatic PPGL. No germline pathogenic variants of *SDHA*, *SDHB*, *SDHC*, *SDHD*, *SDHAF2*, *MAX*, *KIF1B*, *TMEM127*, *DLST*, and *SLC25A11* were detected in the peripheral blood samples. Testing for *RET*, *VHL*, and *NF1* were not included in the panel; however, the patient had no syndromic clinical manifestations, raising suspicions for multiple endocrine neoplasia type 2A/2B, von-Hippel Lindau disease, and neurofibromatosis type 1.

**Table 1.** Laboratory findings of the patient

Parameters	At diagnosis	4 months
WBC	7970/µL	12 310/µL
Hb	13.2 g/dL (132 g/L)	9.1 g/dL (91 g/L)
Plt	40.0 × 10^4^/µL	44.5 × 10^4^/µL
γGTP	96 IU/L	169 IU/L
AST	37 IU/L	47 IU/L
ALT	33 IU/L	42 IU/L
LDH	345 IU/L	982 IU/L
Alb	4.0 g/dL (40 g/L)	1.2 g/dL (12 g/L)
Cre	0.60 mg/dL (53.4 mmol/L)	0.36 mg/dL (31.8 mmol/L)
FPG	126 mg/dL (6.99 mmol/L)	91 mg/dL (5.05 mmol/L)

Abbreviations: γGTP, gamma glutamyl transferase; Alb, albumin; ALT, alanine aminotransferase; AST, aspartate aminotransferase; Cre, creatinine; FPG, fasting plasma glucose; Hb, hemoglobin; LHD, lactate dehydrogenase; Plt, platelet; WBC, white blood cell.

**Table 2.** Changes in the urinary catecholamines during the course

Parameters	Day 1	Day 3	1 month	3 months	4 months	Reference range
DA	53 000 µg/day	66 000 µg/day	150 000 µg/day	230 000 µg/day	180 000 µg/day	365-961.5 µg/day
	258 322 nmol/day	321 684 nmol/day	731 100 nmol/day	1 121 020 nmol/day	877 320 nmol/day	1779-4686 nmol/day
NA	1690 µg/day	1970 µg/day	1710 µg/day	3800 µg/day	4430 µg/day	48.6-168.4 µg/day
	9989 nmol/day	11 644 nmol/day	10 107 nmol/day	22 461 nmol/day	26 185 nmol/day	287-995 nmol/day
Ad	355 µg/day	432 µg/day	476 µg/day	1500 µg/day	2270 µg/day	3.4-26.9 µg/day
	1937 nmol/day	2358 nmol/day	2598 nmol/day	8188 nmol/day	12 391 nmol/day	318-146 nmol/day
MN	2.9 mg/day	3.2 mg/day	3.9 mg/day	15 mg/day	28 mg/day	0.0-0.19 mg/day
	14 703 nmol/day	16 224 nmol/day	19 773 nmol/day	76 052 nmol/day	141 965 nmol/day	0-963 nmol/day
NMN	8.6 mg/day	9.0 mg/day	12 mg/day	34 mg/day	42 mg/day	0.09-0.33 mg/day
	46 942 nmol/day	49 125 nmol/day	65 500 nmol/day	185 585 nmol/day	229 252 nmol/day	491-1801nmol/day

All data are evaluated using a 24-hour urine storage with HCl addition.

Abbreviations: Ad, adrenaline; DA, dopamine; MN, metanephrine; NMN, normetanephrine; NA, noradrenaline.

To better understand the characteristics of the present case from the context of the wider literature, we reviewed 18 previously published cases of DA-producing PPGL in 12 reports [3-14], together with patients who visited Kanazawa University Hospital from 2022 to 2023 for follow-up after PPGL resection. Data on the origin of the PPGL, age at diagnosis, sex, and catecholamine profile at the diagnosis were collected. Patients who did not undergo catecholamine profiling were excluded. A total of 34 patients of PPGL were recruited from the Kanazawa University Hospital.

**Figure 3.**
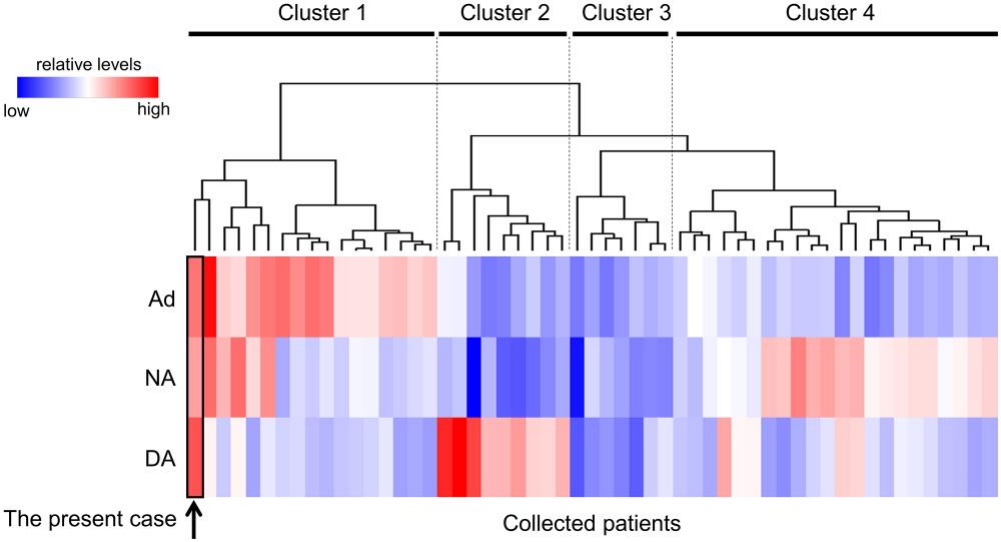
The hierarchical clustering analysis based on the catecholamine profiles. The data of 18 patients reported as DA-producing PPGL in the literature and 34 patients followed-up in Kanazawa University were included. Each patient and catecholamines were aligned horizontally and vertically, respectively. Dashed lines separate each cluster according to hierarchical clustering analysis. The colors in the heatmap represent relative levels of urinary Ad, NA, or DA excretion. Abbreviations: Ad, adrenaline; DA, dopamine; NA, noradrenaline; PPGL, pheochromocytoma/paraganglioma.

Hierarchical clustering analysis was used to highlight the unique catecholamine production of the present case using R (version 4.2.2). Patients were categorized into 4 clusters based on the catecholamine profiling (Fig. 3): patients in cluster 1 had high Ad levels and normal DA levels, patients in cluster 2 showed high DA levels and normal Ad levels, and patients in clusters 3 and 4 showed no catecholamine elevation and high NA levels, respectively. The characters of each cluster are presented in Table 3. The patients in cluster 2 showed significantly larger nodule and higher frequency in which the PPGL arising from the extra-adrenal area than those in other clusters (Table 3). The present case was categorized into cluster 1. There were no other case concomitantly elevating Ad, NA, and DA levels, except for the present case.

**Table 3.** Characteristics of each cluster

	Cluster 1	Cluster 2	Cluster 3	Cluster 4
Number	17	9	7	22
Extra-Ad	5%	88%	57%	40%
Age at diagnosis, y	68 (49-75)	61 (48-70)	50 (34-66)	54 (50-65)
% female	47%	77%	42%	54%
u-DA	1000 (730-1500) μg/day	7933 (7291–72 063) μg/day	420 (315-757) μg/day	1005 (712-1662) μg/day
	4874 (3558-7311) nmol/day	38 665 (35 536–351 235) nmol/day	2047 (1535-3689) nmol/day	4898 (3470-8100) nmol/day
u-NA	233 (168-1195) μg/day	68 (40-122) μg/day	67 (63-109) μg/day	541 (316-963) μg/day
	1377 (993-7063) nmol/day	401 (236-721) nmol/day	396 (372-644) nmol/day	3197 (1867-5692) nmol/day
u-Ad	94.7 (64.4-327.0) μg/day	6.4 (4.0-12.8) μg/day	5.2 (2.4-8.3) μg/day	12.1 (7.4-14.6) μg/day
	516 (351-1785) nmol/day	34 (21-69) nmol/day	28 (13-45) nmol/day	66 (40-79) nmol/day
Size	39 (28-62) mm	104 (70-118) mm	28.5 (19-67) mm	62 (35-93) mm

All data are expressed as median and interquartile range.

Abbreviations: Ad, adrenaline; DA, dopamine; Extra-Ad; origin in the extra-adrenal area; NA, noradrenaline; u-Ad, urinary adrenaline excretion; u-DA, urinary dopamine excretion; u-NA, urinary noradrenaline excretion.

## Treatment

The α-1 blocker, doxazosin, was administered and titrated to a dose of 16 mg/day. Although a debulking resection of the adrenal mass was raised [1, 15], adrenalectomy was refused for following reasons: (1) the inferior vena cava was infiltrated by the adrenal mass; (2) severe catecholamine symptoms, such as heart failure or malignant hypertension, were absent; and (3) liver nodules may contribute to elevated catecholamine levels, even if adrenalectomy is performed. Chemotherapy with cyclophosphamide, vincristine, and dacarbazine was begun. Acetaminophen, naproxen, and hydromorphone were administered to treat abdominal pain and pyrexia.

## Outcome and Follow-up

Despite 4 months of chemotherapy, the adrenal mass progressed to 188 mm in size and the liver nodules proliferated with hepatomegaly. The levels of the urinary excretions of Ad, NA, DA, metanephrine, and normetanephrine increased to 2270 µg/day (12 391 nmol/day), 4430 µg/day (26 185 nmol/day), 180 000 µg/day (877 320 nmol/day), 28 mg/day (141 965nmol/day), and 42 mg/day (229 252 nmol/day), respectively (Table 2). Subsequently, the patient was placed under hospice care.

## Discussion

The case presented in this report is unique in that it shows overproduction of multiple catecholamines (Ad, NA, and DA) in metastatic PPGL with adrenal mass and liver nodules. Theoretically, the NA and Ad levels in DA-producing PPGL are relatively normal because DBH dysfunction or immature catecholamine vesicles fails to produce downstream products, such as NA and Ad [3, 4]. In fact, no cases of PPGL with the concomitant elevation of DA, NA, and Ad levels have been previously reported.

In the present case, the ^123^I-MIBG scintigraphy and FDG positron emission tomography (FDG-PET) findings suggested internodular heterogeneity among the right adrenal mass, liver nodules in the right lobe, and liver nodules in the left lateral segment. The signal intensity of a disease-specific radioisotope generally depends on receptor or transporter levels on tumors. These receptor and transporter gene/protein alterations are observed in poorly differentiated or dedifferentiated metastatic tumors and also affect hormone production, especially in endocrine organs [16, 17]. For example, malignant neuroendocrine tumors occasionally show low uptake on somatostatin scintigraphy and high uptake on FDG-PET [18, 19] because of SSTR2 deficiency and high glucose transporter [19, 20] or modified glucose metabolism, respectively. Interestingly, the differentiation between primary and metastatic nodules occasionally differs. Several reports have shown that metastatic tumors have the potential to exhibit hormone secretion patterns that are different from those of the primary tumor [21-23]. Therefore, the internodular heterogeneity of nuclear imaging findings could suggest that the malignant grades and hormone profiles differ among the nodules. Based on these observations, we hypothesized that DBH or catecholamine vesicles were impaired solely in metastatic nodules in the right liver and promoted DA overproduction, whereas DBH function was preserved in the primary adrenal mass to produce NA and Ad. In addition, catecholamine production and ^123^I-MIBG uptake in the left liver nodules may differ from those in the primary adrenal mass. This internodular heterogeneity noted on nuclear imaging may reflect the different catecholamines produced by the adrenal mass vs the metastatic foci, thereby resulting in the elevation of multiple catecholamine (DA, NA, and Ad). Immunohistochemistry could help provide more conclusive evidence regarding the above hypothesis.

Hierarchical clustering analysis based on catecholamine profiles was conducted in this study. To the best of our knowledge, no study has previously performed clustering analysis using clinical parameters in cases of PPGL. The clustering analysis used in this study explicitly separated DA-, NA-, and Ad-dominant types. Cluster 1 represented the Ad-dominant type and cluster 2 represented the DA-dominant type, whereas clusters 3 and 4 represented the nonfunctioning and NA-dominant types, respectively. The DA-dominant cluster frequently comprised female patients and the PPGL arose from the extra-adrenal area. Previous reports have suggested that female patients with PPGL have a higher proportion of head and neck PPGL than male patients [24]. Catecholamine profiles and aggressiveness of PPGL may be determined by genetic and epigenetic alteration, as well as sex and ancestry. This case was categorized as an Ad-dominant cluster. According to clustering analysis, Ad elevation was the most characteristic phenotype in the present case. Although several patterns of clustering analysis were performed, the case presented in this report was not included in the DA-dominant type or independent cluster. This clustering analysis is based on the “relative” levels of each parameter (Ad, NA, or DA). Thus, the elevation of 2 parameters (Ad and NA) might influence the clustering than 1 parameter (DA). Unfortunately, the patient had a poor prognosis. Our clustering analysis required additional clinical information regarding prognosis, family history, metastasis, and germline pathogenic variants. This improved clustering system could help to predict the clinical course of newly diagnosed patients.

This study has several limitations. First, histology, gene and protein expression patterns, and somatic pathogenic variants, such as succinate dehydrogenase, could not be evaluated in the adrenal and liver nodules because of a lack of pathology specimens; this was due to the patient being managed nonsurgically. Second, our review lacked data on metanephrine and normetanephrine, which are superior to urinary Ad and NA for evaluation of catecholamine hyperproduction. Third, germline pathogenic variants of *RET*, *VHL*, and *NF1*, which cause syndromes including PPGL, were not analyzed. Although we cannot precisely deny these pathogenic variants, the patient presented in this report did not show any characteristics of these phenomena or diseases.

To the best of our knowledge, this is the first to report on a case of PPGL with concomitant elevation of Ad, NA, and DA. This case report also describes the internodular heterogeneity between primary and metastatic foci on nuclear imaging with ^123^I-MIBG and FDG-PET, which may suggest the existence of varying grades of differentiation and resultant catecholamine secretion. However, further studies involving immunohistochemistry are needed to confirm this hypothesis.

## Leaning Points

- Aberrant elevation of DA levels in PPGL may indicate poorly differentiated and/or extra-adrenal PPGL.
- Concomitant elevation of DA, NA, and Ad suggests the possibility that PPGL has multiple foci.
- In metastatic PPGL, the metastasis possibly may dedifferentiate and lose its ability to produce Ad or even NA.

## Data Availability

Some datasets generated and analyzed during the current study are not publicly available, but are available from the corresponding author on reasonable request.
